# Natural Products Targeting on Oxidative Stress and Inflammation: Mechanisms, Therapies, and Safety Assessment

**DOI:** 10.1155/2018/6576093

**Published:** 2018-01-09

**Authors:** Zhenquan Jia, Pon Velayutham Anandh Babu, Wei Chen, Xiaolun Sun

**Affiliations:** ^1^Department of Biology, University of North Carolina at Greensboro, Greensboro, NC 27402, USA; ^2^Department of Nutrition and Integrative Physiology, College of Health, University of Utah, Salt Lake City, UT 84112, USA; ^3^Department of Food Science and Nutrition, National Engineering Laboratory of Intelligent Food Technology and Equipment, Key Laboratory for Agro-Products Postharvest Handling of Ministry of Agriculture, Zhejiang Key Laboratory for Agro-Food Processing, Fuli Institute of Food Science, Zhejiang University, Hangzhou 310058, China; ^4^The Center of Excellence for Poultry Science, Dale Bumpers College of Agricultural, Food & Life Sciences, University of Arkansas, Fayetteville, AR 72701, USA

Substantial evidence suggests that overproduction of reactive oxygen species (ROS) and its associated oxidative stress and inflammatory responses play a crucial role in the pathogenesis of various human chronic diseases including diabetes, cardiovascular diseases, obesity, neurodegenerative diseases, and cancer. Enhanced ROS production can cause oxidative damage to the cellular macromolecules including lipids, proteins, and DNA. In addition, oxidative stress activates nuclear factor kappa B (NF-*κ*B), nuclear factor-erythroid 2-related factor 2 (Nrf2), and antioxidant-responsive elements (ARE) to regulate anti-inflammatory and antioxidative genes involved in inflammatory responses and cellular defense mechanisms. Recent studies have showed that certain natural compounds and/or their derived small molecules have the ability to protect cells from oxidative stress and ameliorate various oxidative stress-related diseases. However, a large number of natural products are underutilized and unexploited because their bioactivities are unknown and the molecular mechanisms involved are yet to be identified. Understanding the mechanisms of actions of natural products would shed further light into the application of these compounds in the prevention and treatment of oxidative stress-related diseases in humans. This special issue includes two invited reviews and twelve original research articles that are focused on the antioxidative/anti-inflammatory effects of various natural compounds and their safety in vitro and in vivo. Further, these studies identified the cellular and molecular mechanisms underlying the bioactivities of these natural compounds.

Hongjingtian is a botanical drug manufactured from the *Rhodiola allichiana* var. *cholaensis* extract which is used for the prevention and treatment of cardiovascular diseases. However, the molecular mechanisms involved are unknown. The paper by S. Zhang et al. characterized the cardioprotective effects of Hongjingtian using hypoxia/reoxygenation- (H/R-) induced myocardial injury in H9c2 cardiomyocytes and myocardial ischemia-injured rats. They demonstrated that the administration of Hongjingtian significantly decreases myocardial injury and improves the cardiac function in myocardial ischemia-injured rats. Further, the protective mechanisms of Hongjingtian are mediated via the activation of AKT/beclin-1, AKT, and ERK/mTOR signaling pathways which is associated with an enhancement of the antioxidant system, inhibition of apoptosis, and promotion of autophagy in cardiomyocytes.

Luteolin-6-C-neohesperidoside (5,7,3′,4′-tetrahydroxy-6-C-neohesperidose) is a flavone glycoside isolated from moso bamboo leaf. The article by F. Duan et al. investigated the antifatigue activity of luteolin-6-C-neohesperidoside by evaluating muscle and liver functional activities in an experimental rat model subjected to a weight-loaded forced swimming test (FST). The results showed that the administration of luteolin-6-C-neohesperidoside significantly improves the endurance of rats in the FST suggesting an antifatigue effect of this compound. Oxidative stress has been implicated in the fatigue-related disorders. This study further demonstrated that luteolin-6-C-neohesperidoside possesses an antioxidant activity, reduces lipid peroxidation, and protects cells from oxidative stress injury in the liver and skeletal muscle of rats after exhaustive exercise. The authors further showed that the underlying mechanism of the antifatigue effect of luteolin-6-C-neohesperidoside is mediated by the activation of the Nrf2/ARE signaling pathway to modulate the inflammatory response.

The paper by W. Chen et al. investigated the protective effect and mechanisms of the mulberry fruit extract against ethyl carbamate-induced cytotoxicity in human liver HepG2 cells. Ethyl carbamate is a known genotoxic carcinogen that is found in different alcoholic beverages, fermented food products, and tobacco. This study showed that the mulberry fruit extract exhibits an antioxidant capacity and protects human liver HepG2 cells against ethyl carbamate cytotoxicity by decreasing reactive oxygen species, increasing cellular antioxidant glutathione, and improving collapse of mitochondrial membrane potential. The authors concluded that the mulberry fruit extract is able to afford protection against ethyl carbamate-induced cytotoxicity due to its antioxidant nature.

Methane is commonly used as a form of natural gas. This gas is mostly produced in the large intestine by the anaerobic flora in the human gastrointestinal tract. The paper by W. Wang et al. investigated the protective effects of methane-rich saline on spinal cord injury. The authors showed that administration of methane-rich saline repairs spinal cord injury in Sprague-Dawley rats. In addition, they found that the protective effect of methane is mediated through the inhibition of activated microglia-mediated oxidative stress, inflammatory cytokines, and cell apoptosis. Consistent with these results, the paper by A. Sun et al. demonstrated that methane-rich saline protects against lipopolysaccharide-induced acute lung injury mainly due to its potential antioxidative, anti-inflammatory, and antiapoptotic activities.

2,3,5,4′-Tetrahydroxystilbene-2-O-*β*-D-glucoside (TSG) is an active component in the Chinese herb *Polygonum multiflorum*. The paper by W. Yu et al. demonstrated the protective effect of TSG on lipopolysaccharide- (LPS-) induced proinflammatory cytokine production in murine macrophage RAW264.7 cells. The authors showed that the protective mechanism of TSG is mediated through the NRF2-ARE pathway that regulates the antioxidant genes such as heme oxygenase-1, Mn-superoxide dismutase, catalase, NADP(H) quinone oxidoreductase 1, and glutathione peroxidase-1.

The cell wall extracts of *Saccharomyces cerevisiae* yeast (SCCWE) have been considered valuable natural immunostimulants. As it is rich in *β*-glucan, SCCWE has been suggested to be a good source of prebiotics. The paper by G. Liu et al. investigated the effects of dietary SCCWE on animal growth performance and diarrhea in weaned piglets. In this study, dietary SCCWE improved plasma essential and nonessential amino acid levels, intestinal morphology, and animal growth performance. The authors concluded that SCCWE is safe and effective in reducing diarrhea. Accordingly, they suggested that this product can be a good candidate to be a substitute for antibiotics in weaned piglets.

Astaxanthin (AST; 3,30-dihydroxy-b,b-carotene-4,40-dione) is a carotenoid. But unlike other carotenes, AST is not converted into vitamin A in vivo. AST possesses antioxidant activity higher than that of *β*-carotene and *α*-tocopherol. The paper by F. Harada et al. investigated the effect of oral nano-AST on UV-induced photokeratitis in mice. In this study, the administration of 50 mg/kg nano-AST preserved epithelial morphology and significantly decreased the number of TUNEL- and NF-*κ*B-positive cells in the cornea with respect to the UVB control group. In addition, nano-AST reduced inflammation and decreased cell death in corneal tissue as measured by immunohistochemistry, Western blot analysis, and qPCR. Also, there were no obvious adverse side effects observed in the animals of the nano-AST group. The authors concluded that nano-AST is effective in protecting the ocular surface against the detrimental effects of acute UVB exposure.

*Angelica sinensis* (Oliv.) Diels (Umbelliferae) (AS) is a well-known traditional Chinese medicine for treating gynecological diseases and replenishing blood. *Angelica sinensis* polysaccharide (ASP) is isolated and purified from the root of *A. sinensis*. The paper by K. Wang et al. extracted ASP from the root of *A. sinensis* and examined the therapeutic efficacy of ASP against anemia of chronic disease (ACD) in a well-established rat model induced by complete Freund's adjuvant (CFA). In this study, the administration of ASP increased ferroportin expression, mobilized iron from the liver and spleen serum iron levels, and relieved the anemia by blocking the IL-6/STAT3 and BMP/SMAD pathways. The authors concluded that ASP could be a potential option for the treatment of patients suffering from ACD.

Anthocyanins are polyphenolic phytochemicals rich in a wide range of berries. Studies showed that cardiovascular benefits of red wine are due to anthocyanins, particularly delphinidin. The paper by K. Goszcz et al. examined the protective effects of delphinidin at biologically relevant concentrations on cultured endothelial cells. This study showed that delphinidin rapidly degrades at physiological conditions (t1/2~30 min) generating gallic acid. As a major degradation product, gallic acid shared many of the antioxidant protective characteristics of the delphinidin including an increase in total intracellular GSH. At subtoxic concentrations, both delphinidin and gallic acid can protect against oxidative stress induced by various prooxidants through a mechanism that is associated with increased glutathione. The authors concluded that delphinidin is intrinsically unstable at biologically relevant concentrations, and thus, the protective effects of delphinidin might not be due to its direct antioxidant activity in vivo.

Salidroside is a bioactive compound isolated from *Rhodiola rosea*. As a traditional Chinese medicine, *Rhodiola rosea* has been widely used for the treatment of inflammation, cancer, and neurodegenerative diseases, but its underlying mechanisms remain unclear. High-mobility group box 1 (HMGB1) is a highly conserved nonhistone DNA-binding protein, and it plays a crucial role in a severe form of systemic inflammation known as sepsis. The paper by Z. Qi et al. examined the effects of salidroside on HMGB1 release both in vitro and in an experimental rat model of sepsis induced by caecal ligation and puncture (CLP). This study found that salidroside inhibits HMGB1 release in LPS-induced RAW264.7 cells by suppressing LPS-induced HMGB1 acetylation. Salidroside also reduced the serum HMGB1 level in CLP rats by regulating the AMPK-SirT1 signaling pathway. These results suggested that salidroside could be considered a potential treatment for sepsis by targeting on HMGB1.

Huang-Lian-Jie-Du-Decoction (HLJDD) is a popular prescription of traditional Chinese medicine for the treatment of ischemic stroke. Berberine, baicalin, and jasminoidin are three major active ingredients of HLJDD. The paper by Q. Zhang et al. examined the effect of these compounds on brain injury after focal cerebral ischemia in a rat model. In this study, berberine, baicalin, and jasminoidin ameliorated abnormal metabolism and reduced oxidative stress, neuron autophagy, and inflammatory response. The authors concluded that these three compounds are responsible for the protective effect of HLJDD on ischemic stroke.

The review article by V. M. Thadhani and V. Karunaratne introduced the recent progress regarding the antidiabetic potential of lichens. Lichens are a potential natural source of bioactive compounds. The authors highlighted the recent findings on the antidiabetic effects of lichen extracts reported both in vitro and in vivo. This review particularly emphasizes that lichen extracts could be an excellent inhibitor for *α*-amylase and *α*-glucosidase to exert their antidiabetic effects.

H.-L. Wang et al.'s review article evaluated the recent findings on the neuroprotective effects of astragaloside IV on experimental ischemic stroke and discussed the molecular mechanisms involved. Astragaloside IV is a major component of *Radix Astragali seu Hedysari* (Huangqi), an important herb commonly used in traditional Chinese medicine. The authors used eight databases including Web of Science, Chinese Biomedical Literature Database, and PubMed to search the articles related to this topic from inception to March 2016. Based on their evaluation, they conclude that astragaloside IV improves neurological deficits and reduces the blood-brain barrier permeability in experimental cerebral ischemia which is mediated through the antioxidant, anti-inflammatory, and antiapoptosis properties of astragaloside IV.

In summary, this special issue covers a series of topics addressing the antioxidative/anti-inflammatory effects of various natural products and their roles in the prevention and treatment of chronic diseases. The new findings presented in this special issue would enrich our understanding on the efficacy and safety of these natural products for future therapeutic drug development. With that, we would like to thank the authors for their contributions to this special issue and the reviewers as well as staff editorial for their dedication and hard work.

*Zhenquan Jia* *Pon Velayutham Anandh Babu* *Wei Chen* *Xiaolun Sun*

